# Clinical Characteristics and Outcome Between Gallbladder Squamous Cell Carcinoma and Adenocarcinoma: A Propensity Matched Analysis Based on the Surveillance, Epidemiology, and End Results Database

**DOI:** 10.3389/fonc.2022.833447

**Published:** 2022-05-02

**Authors:** Xiaorong Chen, Yuwen Zhou, Qian Xu, Dan Pu, Xinyao Shu, Guixia Wei, Meng Qiu

**Affiliations:** ^1^ Department of Abdominal Cancer, Cancer Center, West China Hospital of Sichuan University, Chengdu, China; ^2^ Department of Biotherapy, Cancer Center, West China Hospital of Sichuan University, Chengdu, China; ^3^ West China Medical Publishers, West China Hospital of Sichuan University, Chengdu, China; ^4^ Lung Cancer Center, West China Hospital of Sichuan University, Chengdu, China

**Keywords:** squamous cell carcinoma, adenocarcinoma, propensity matching, SEER, outcome

## Abstract

**Background:**

Gallbladder squamous cell carcinoma (GSCC) is a rare carcinoma with limited evidence in literature, making it particularly difficult to study. Surveillance, Epidemiology, and End Results Database (SEER) were used to stress the clinicopathological features and outcomes associated with this tumor.

**Methods:**

SEER registries were used to identify GSCC and gallbladder adenocarcinoma (GAC) cases from 2004 to 2015. The Propensity matching (PSM) method was used for minimized potential difference between the two types and the utmost. Patients with GSCC versus GAC were compared using the clinicopathological features and outcomes.

**Results:**

There were 121 patients with GSCC and 6 580 patients had GAC. Compared with the GAC cohort, the GSCC cohort had a lower proportion of well-differentiated histology (3.3% vs. 12.1%, *p* < 0.001) and was diagnosed at a later T-stage (*p* < 0.001). Regarding treatment, patients treated with surgery, chemotherapy or radiation were associated with significantly better outcome than patients without undergoing these treatment modalities. In both univariate and multivariate analyses, GSCC histology was associated with worse prognosis than GAC histology.

**Conclusions:**

Patients with GSCC were associated with a worse outcome than the GAC cohort. The independent risk factors for patients with GSCC are surgery and chemotherapy.

## Introduction

Gallbladder carcinoma (GBC), a relatively uncommon malignancy, accounts for 4% of all gastrointestinal tract neoplasms (1). Most of the patients usually do not exhibit clinical symptoms or show some atypical symptoms with a 5-year survival rate of less than 10% (2, 3). About 97% of the diagnosed GBC in these patients is adenocarcinoma (AC), and its clinicopathological features and survival outcomes are relatively well-studied. Gallbladder squamous cell carcinoma (GSCC) is rarer, accounting for about 3%, which is unrecognized (4, 5). According to previous reports, squamous cell carcinoma variants of different anatomic sites exhibit completely different biology and clinical outcome compared to adenocarcinoma, depending on the primary tumor origin. For example, squamous cell carcinoma (SCC) of the cervix exhibit better results when compared with AC (6).

To date, most literatures regarding GBC consists of case reports/small case series, and very few studies have compared AC to SCC (7, 8). As with AC, surgical resection is the optimal therapeutic modality in patients with GSCC. And, patients with GSCC, diagnosed at early-stage, usually undergo simple cholecystectomy. In contrast, cholecystectomy, partial hepatectomy, and portal lymphadenectomy were usually conducted in patients with progressive disease (8–10). Regarding chemotherapy, there remains a controversy; some studies indicated that GSCC was resistant to chemotherapy, while others demonstrated that patients with GSCC can benefit from adjuvant radio-chemotherapy (8, 11, 12).

To better understand the clinicopathological features, treatment modalities, and survival of GSCC, especially comparing with gallbladder adenocarcinoma (GAC), we used Surveillance, Epidemiology, and End Results (SEER) Database (1975–2016) to provide comprehensive recognition concerning GSCC and survival outcomes between GSCC and GAC.

## Material and Methods

### Patients

All data of patients with GSCC and GAC were obtained from the SEER database 18 Regs (with additional treatment fields, 1975–2016 varying), using the SEER*Stat software (version 8.3.6; Surveillance Research Program, NCI, Bethesda, MD). Additional selection criteria for the SEER*Stat software used in identifying gallbladder cancer were as follows: 1) “Gallbladder” (C23.9) was limited to the site and 2) The histological subtype was defined as SCC and adenocarcinoma (AC) according to the International Agency for Research on Cancer classification using the International Classification of Diseases for Oncology, 3rd edition (ICD-O-3) histology codes. The ICD-0-3 histology codes of histological subtypes were categorized into SCC (8070–8075) and AC (8140, 8144, 8310, 8480, and 8490). Patients diagnosed with more than one malignancy and those with information completely absent were excluded.

### Variables

The following clinicopathological information (age, sex, race, marital status, SEER stage, grade, TNM stage, surgery, radiation, chemotherapy, survival months, and vital status) was obtained from each patient. The age at diagnosis was divided into three groups: ≤40, 40–60, and >60. All patients were staged on account of the SEER stage (localized, regional, and distant) and American Joint Commission on Cancer (AJCC) 6th Edition Staging System. Overall survival (OS) was defined as the time from confirmation diagnosis to death of any cause. Disease-specific-free survival (DSS) referred to the interval from definite diagnosis to death of these tumors.

### Statistical Analysis

All categorical variables were presented with percentages. The χ2 test was used to compare demographic, clinical characteristics, and treatment of patients with GSCC/GAC. Because the uneven baseline features may potentially impact survival outcomes, a 1:2 propensity matching (PSM) method was used for removing the baseline variation to the utmost. The variables used for matching were age, sex, race, marital status, SEER stage, grade, t, n, and m stage, the metastatic status of bone, brain, liver, and lung, surgery, radiation, and chemotherapy. To ensure suitable matches, the nearest neighbor matching algorithm without replacement was used. Cumulative survival curves were shown using the Kaplan–Meier method, and the log-rank test was used to compare differences. Univariate and multivariate survival analyses were conducted using the Cox proportional hazard model, and the hazard ratios (HRs) with 95% confidence intervals (95% CI) were calculated. To determine the HRs in a matched population stratified by covariates, a subgroup analysis of gallbladder cancer were also conducted. All statistical analysis was conducted using R software version 3.6.2 (http://www.R-project.org) and SPSS 25.0 software e (IBM Corp., Armonk, NY, USA). The *p*-values < 0.05 were considered statistically significant.

## Results

### Demographic and Clinical Characteristics

**Figure 1:**
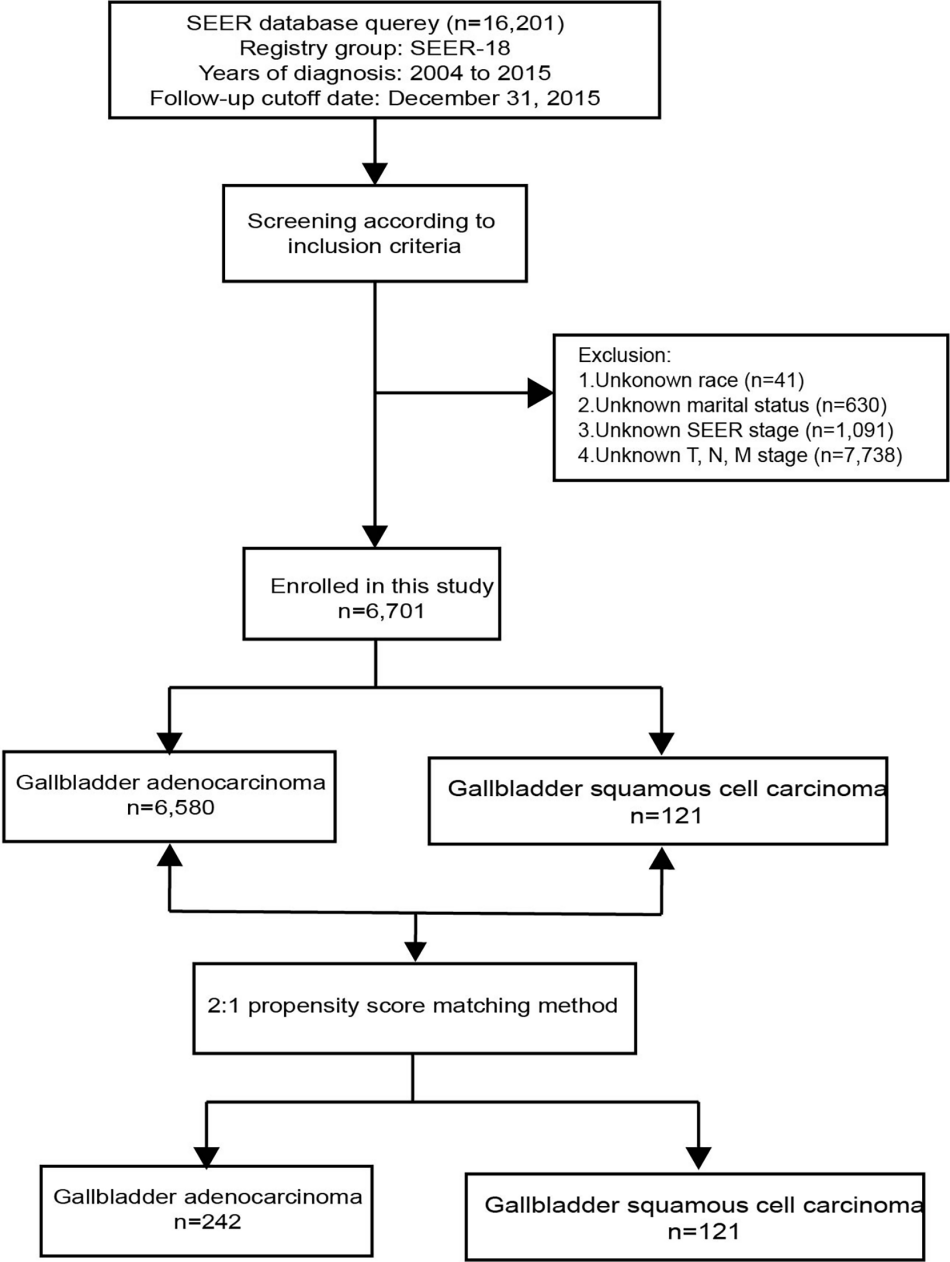
Flow chart.

A total of 16,201 patients selected in the SEER database were eligible for inclusion from January 1^st^, 2004–December 31^st^, 2015, in this study. Ultimately 6,701 patients were selected for the final data analysis; 9,500 patients were excluded according to the predefined exclusion criteria (**Figure 1**). Among 6,701 patients, 121 (1.81%) had GSCC and 6,580 (98.19%) had GAC. After PSM, there were 121 patients with GSCC and 242 with GAC. Demographic and clinicopathological features of all eligible patients are described (**Table 1**). Similar to GAC patients, most GSCC patients were Caucasian and elderly females (>60 years old). The proportion of females was significantly higher in GAC patients than in GSCC patients (56.2% vs. 69.7%, *p* = 0.002). GSCC were likely to have a distant-stage disease. In contrast, a localized-stage disease was common in GAC patients. Compared to patients with GAC, patients with GSCC had a lower proportion of well-differentiated histology (3.3% vs. 12.1%, *p* < 0.001). T3 and T4 stage diseases were more common in GSCC patients, while GAC patients were usually diagnosed with T2 and T3 stage diseases. Regarding treatment, the use of surgery was significantly lower in GSCC cohorts than in GAC cohorts (57.0% vs. 80.7%, P < 0.001). Radiation and chemotherapy did not show significant differences between the two groups. No statistical significance was observed in other variables like age, race, marital status, N stage, M stage, bone, brain, liver, or lung metastases. There was no significant difference in clinicopathological features after 1:2 matching between GSCC and GAC patients.

**Table 1:** Demographics and clinicopathological characteristics of GSCC and GAC patients.

Variables	Data before PSM			Data after PSM		
	GSCC N = 121	GAC N = 6,580	*p*-value	GSCC N = 121	GAC N = 242	*p-*value
**Age**			0.956			0.833
Age (≤40)	2 (1.7)	97 (1.5)		2 (1.7)	5 (2.1)	
Age (40-60)	26 (21.5)	1,355 (20.6)		26 (21.5)	46 (19.0)	
Age (>60)	93 (76.9)	5,128 (77.9)		93 (76.9)	191 (78.9)	
**Sex**			**0.002**			0.851
Male	53 (43.8)	1,996 (30.3)		53 (43.8)	102 (42.1)	
Female	68 (56.2)	4,584 (69.7)		68 (56.2)	140 (57.9)	
**Race (%)**			0.997			0.772
White	93 (76.9)	5,038 (76.6)		93 (76.9)	191 (78.9)	
Black	15 (12.4)	825 (12.5)		15 (12.4)	24 (9.9)	
Other	13 (10.7)	717 (10.9)		13 (10.7)	27 (11.2)	
**Marital status (%)**			0.532			0.631
Married	102 (84.3)	5,703 (86.7)		102 (84.3)	210 (86.8)	
Unmarried	19 (15.7)	877 (13.3)		19 (15.7)	32 (13.2)	
**SEER Stage (%)**			**0.003**			0.556
Localized	30 (24.8)	2,583 (39.3)		30 (24.8)	59 (24.4)	
Regional	41 (33.9)	1,605 (24.4)		41 (33.9)	70 (28.9)	
Distant	50 (41.3)	2,392 (36.4)		50 (41.3)	113 (46.7)	
**Grade (%)**			**<0.001**			0.354
Well differentiated	4 (3.3)	795 (12.1)		4 (3.3)	11 (4.5)	
Moderately differentiated	42 (34.7)	2,383 (36.2)		42 (34.7)	65 (26.9)	
Poorly differentiated	35 (28.9)	2,185 (33.2)		35 (28.9)	62 (25.6)	
Undifferentiated	2 (1.7)	72 (1.1)		2 (1.7)	4 (1.7)	
Unknown	38 (31.4)	1,145 (17.4)		38 (31.4)	100 (41.3)	
**T**			**<0.001**			0.569
1	14 (11.6)	1,017 (15.5)		14 (11.6)	31 (12.8)	
2	18 (14.9)	2,236 (34.0)		18 (14.9)	33 (13.6)	
3	69 (57.0)	2,954 (44.9)		69 (57.0)	150 (62.0)	
4	20 (16.5)	373 (5.7)		20 (16.5)	28 (11.6)	
**N**			0.419			0.713
0	88 (72.7)	4,532 (68.9)		88 (72.7)	170 (70.2)	
1	33 (27.3)	2,048 (31.1)		33 (27.3)	72 (29.8)	
**M**			0.985			0.453
0	85 (70.2)	4,655 (70.7)		85 (70.2)	159 (65.7)	
1	36 (29.8)	1,925 (29.3)		36 (29.8)	83 (34.3)	
**Bone metastasis (%)**			0.483			0.851
No	70 (57.9)	3,572 (54.3)		70 (57.9)	136 (56.2)	
Yes	0 (0.0)	53 (0.8)		–	–	
Unknown	51 (42.1)	2,955 (44.9)		51 (42.1)	106 (43.8)	
**Brain metastasis (%)**			0.788			0.716
No	70 (57.9)	3,618 (55.0)		70 (57.9)	134 (55.4)	
Yes	0 (0.0)	5 (0.1)		0 (0.0)	1 (0.4)	
Unknown	51 (42.1)	2,957 (44.9)		51 (42.1)	107 (44.2)	
**Liver metastasis (%)**			0.135			0.830
No	52 (43.0)	2,964 (45.0)		52 (43.0)	96 (39.7)	
Yes	19 (15.7)	668 (10.2)		19 (15.7)	41 (16.9)	
Unknown	50 (41.3)	2,948 (44.8)		50 (41.3)	105 (43.4)	
**Lung metastasis (%)**			0.800			0.767
No	68 (56.2)	3,497 (53.1)		68 (56.2)	135 (55.8)	
Yes	2 (1.7)	120 (1.8)		2 (1.7)	2 (0.8)	
Unknown	51 (42.1)	2,963 (45.0)		51 (42.1)	105 (43.4)	
**Surgery (%)**			**<0.001**			0.479
No	52 (43.0)	1,270 (19.3)		52 (43.0)	115 (47.5)	
Yes	69 (57.0)	5,310 (80.7)		69 (57.0)	127 (52.5)	
**Radiation therapy (%)**			0.310			0.956
No	106 (87.6)	5,511 (83.8)		106 (87.6)	210 (86.8)	
Yes	15 (12.4)	1,069 (16.2)		15 (12.4)	32 (13.2)	
**Chemotherapy (%)**			1.000			0.731
No	77 (63.6)	4,177 (63.5)		77 (63.6)	148 (61.2)	
Yes	44 (36.4)	2,403 (36.5)		44 (36.4)	94 (38.8)	

>GSCC>, gallbladder squamous cell carcinoma; >GAC>, gallbladder adenocarcinoma; >PSM>, propensity matching. The bold part indicates that the data is statistically significant.

Other include American Indian/Alaskan native, and Asian/Pacific Islander, and others unspecified.

### Prognostic Analysis of Patients With GSCC

**Figure 2:**
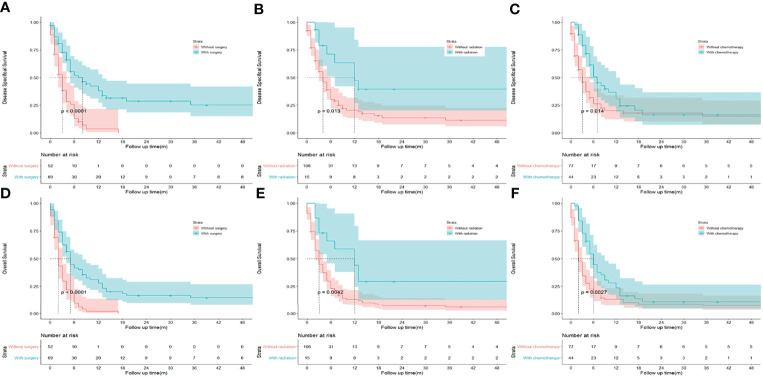
Kaplan-Meier analysis of disease specifical survival and overall survival between patients underwent surgery **(A, D)**, radiation **(B, E)**, and chemotherapy **(C, F)** or not in patients with gallbladder squamous cell carcinoma.

To identify potential predictors of OS in GSCC cohort, univariate and multivariate analyses were conducted regarding different clinicopathological variables. (**Table 2**) In the univariate analysis, the following variables were separately associated with OS: distant-stage, status of metastasis, and liver metastasis. Regarding treatment, there was a significant difference in DSS (median DSS, 8.0 vs. 3.0; HR, 0.347; *p* < 0.001; **Figure 2A**) and OS (median OS, 5.0 vs 2.0; HR, 0.406; *p* < 0.001; **Figure 2D**) in patients treated with or without surgery. Similar trend was observed during chemotherapy (median DSS, 7.0 vs. 3.0; HR, 0.594; *p* = 0.014; **Figure 2C**; median OS, 6.0 vs 2.0; HR, 0.572; *p* = 0.006; **Figure 2F**) and radiation (median DSS, 12.0 vs 4.0; HR, 0.426; *p* = 0.013; **Figure 2B** median OS, 12.0 vs 3.0; HR, 0.421; *p* = 0.010; **Figure 2E**). As regarding to age, sex, race, marital status, grade, T and N stages and lung metastasis did not appear to significantly influence OS of patients with GSCC in both analyses.

**Table 2:** Univariate and multivariate analyses of patients with GSCC.

Characteristics	Univariable	95.0% CI	p value	Multivariable	95.0% CI	*p* value
	Hazard ratio			Hazard ratio		
**Age**						
Age (≤40)	Reference			Reference		
Age (40-60)	1.296	0.305-5.518	0.726	1.349	0.215-8.470	0.749
Age (>60)	1.349	0.331-5.500	0.676	1.432	0.256-8.015	0.683
**Sex**						
Male	Reference			Reference		
Female	1.041	0.711-1.522	0.837	0.953	0.598-1.517	0.838
**Race (%)**						
White	Reference			Reference		
Black	1.033	0.594-1.798	0.907	0.984	0.537-1.803	0.959
Other	1.210	0.658-2.226	0.540	1.391	0.676-2.861	0.370
**Marital status (%)**						
Married	Reference			Reference		
Unmarried	1.278	0.764-2.139	0.351	0.932	0.507-1.716	0.822
**Stage (%)**						
Localized	Reference			Reference		
Regional	1.363	0.813-2.285	0.240	1.693	0.750-3.819	0.205
Distant	2.074	1.250-3.439	**0.005**	1.674	0.592-4.738	0.332
**Grade (%)**						
Well differentiated	Reference			Reference		
Moderately differentiated	0.996	0.353-2.810	0.993	1.212	0.351-4.189	0.761
Poorly differentiated	1.115	0.390-3.188	0.839	1.264	0.362-4.419	0.714
Undifferentiated	1.705	0.308-9.428	0.541	1.357	0.194-9.517	0.759
Unknown	2.411	0.847-6.866	0.099	1.345	0.370-4.898	0.653
**T**						
1	Reference			Reference		
2	0.917	0.420-2.000	0.827	1.541	0.639-3.715	0.336
3	1.295	0.679-2.471	0.432	1.214	0.503-2.932	0.666
4	1.231	0.578-2.622	0.590	0.877	0.310-2.479	0.805
**N**						
0	Reference					
1	1.184	0.775-1.807	0.435	1.062	0.587-1.920	0.842
**M**						
0	Reference					
1	1.873	1.233-2.844	**0.003**	1.071	0.457-2.513	0.874
**Liver metastasis (%)**						
No	Reference					
Yes	1.804	1.031-3.154	**0.039**	1.302	0.592-2.864	0.512
Unknown	0.962	0.636-1.456	0.856	0.976	0.600-1.589	0.923
**Lung metastasis (%)**						
No	Reference			–		
Yes	1.034	0.252-4.246	0.963	–		
Unknown	0.856	0.582-1.259	0.430	–		
**Surgery (%)**						
No	Reference			Reference		
Yes	0.406	0.271-0.609	**<0.001**	0.418	0.209-0.837	**0.014**
**Radiation therapy (%)**						
No	Reference			Reference		
Yes	0.421	0.219-0.811	**0.010**	0.716	0.331-1.551	0.398
**Chemotherapy (%)**						
No	Reference			Reference		
Yes	0.572	0.384-0.853	**0.006**	0.411	0.245-0.690	**0.001**

GSCC, gallbladder squamous cell carcinoma; CI, confidence interval.

The bold part indicates that the data is statistically significant.

### Survival Analysis of Matched Patients

**Figure 3:**
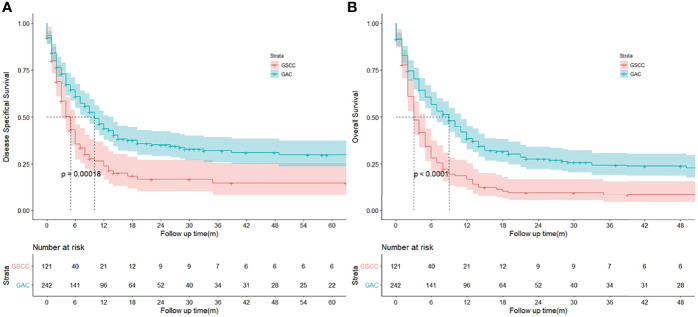
**(A, B)** Kaplan‐Meier curves to compare disease specifical survival (DSS) and overall survival (OS) of patients with gallbladder squamous cell carcinoma (GSCC) and gallbladder adenocarcinoma (GAC), respectively.

After PSM, GSCC was associated with significantly worse OS and DSS compared to GAC. (**Figure 3**) The DSS rates were 23.9% and 14.7% for the GSCC cohort at 1-year and 3-year, respectively, and 43.9% and 32.0% for the GAC cohort (HR, 0.614; 95%CI, 0.469–0.803; *p* < 0.001). Additionally, GSCC patients had 1-year and 3-year OS rates of 16.8% and 8.4%, respectively, while GAC patients were 38.4% and 24.4% (HR, 0.566; 95% CI, 0.444–0.721; *p* < 0.001). A significant difference was detected in the histological subtype, indicating that patients with GSCC presented a poorer outcome than patients with GAC in both univariate and multivariate analyses (**Table 3**).

**Table 3:** Univariate and multivariate analyses of the 1:2 matched cohort (OS).

Characteristics	Univariable	95.0% CI	p value	Multivariable	95.0% CI	p value
	Hazard ratio			Hazard ratio		
**Age**						
Age (≤40)	Reference			Reference		
Age (40-60)	1.320	0.529-3.293	0.552	1.769	0.590-5.309	0.309
Age (>60)	1.311	0.540-3.183	0.550	1.783	0.610-5.214	0.291
**Sex**						
Male	Reference			Reference		
Female	1.214	0.960-1.537	0.106	0.966	0.737-1.267	0.802
**Race (%)**						
White	Reference			Reference		
Black	1.015	0.697-1.479	0.938	1.063	0.708-1.596	0.769
Other	1.181	0.827-1.687	0.361	1.265	0.875-1.831	0.212
**Marital status (%)**						
Married	Reference			Reference		
Unmarried	1.223	0.887-1.687	0.219	1.042	0.734-1.478	0.819
**Stage (%)**						
Localized	Reference			Reference		
Regional	2.848	1.986-4.085	**<0.001**	2.445	1.520-3.932	**<0.001**
Distant	3.863	2.726-5.474	**<0.001**	3.084	1.682-5.654	**<0.001**
**Grade (%)**						
Well differentiated	Reference			Reference		
Moderately differentiated	1.016	0.524-1.971	0.963	1.021	0.516-2.023	0.952
Poorly differentiated	1.451	0.750-2.809	0.269	1.239	0.628-2.445	0.536
Undifferentiated	1.448	0.494-4.242	0.500	1.558	0.510-4.761	0.437
Unknown	2.857	1.495-5.462	**0.001**	1.174	0.571-2.417	0.663
**T**						
1	Reference			Reference		
2	0.650	0.389-1.085	0.100	0.909	0.531-1.559	0.730
3	1.721	1.170-2.531	**0.006**	1.099	0.690-1.749	0.690
4	1.903	1.197-3.028	**0.007**	0.750	0.438-1.286	0.296
**N**						
0	Reference					
1	1.362	1.061-1.747	**0.015**	1.014	0.730-1.407	0.935
**M**						
0	Reference					
1	2.007	1.573-2.560	**<0.001**	1.022	0.644-1.623	0.927
**Liver metastasis (%)**						
No	Reference			Reference		
Yes	1.881	1.347-2.627	**<0.001**	1.137	0.733-1.765	0.566
Unknown	1.198	0.923-1.554	0.176	0.613	0.060-6.317	0.681
**Lung metastasis (%)**						
No	Reference			Reference		
Yes	1.150	0.426-3.107	0.783	0.606	0.211-1.741	0.352
Unknown	1.016	0.803-1.285	0.896	1.750	0.172-17.748	0.636
**Surgery (%)**						
No	Reference			Reference		
Yes	0.291	0.226-0.375	**<0.001**	0.372	0.251-0.551	**<0.001**
**Radiation therapy (%)**						
No	Reference			Reference		
Yes	0.583	0.405-0.838	**0.004**	0.887	0.575-1.369	0.589
**Chemotherapy (%)**						
No	Reference			Reference		
Yes	0.810	0.638-1.028	0.083	0.523	0.389-0.704	**<0.001**
**Histological subtype**						
GSCC	Reference			Reference		
GAC	0.566	0.444-0.721	**<0.001**	0.487	0.379-0.628	**<0.001**

GSCC, gallbladder squamous cell carcinoma; GAC, gallbladder adenocarcinoma; OS, overall survival.

The bold part indicates that the data is statistically significant.

### Stratified Analysis

**Figure 4:**
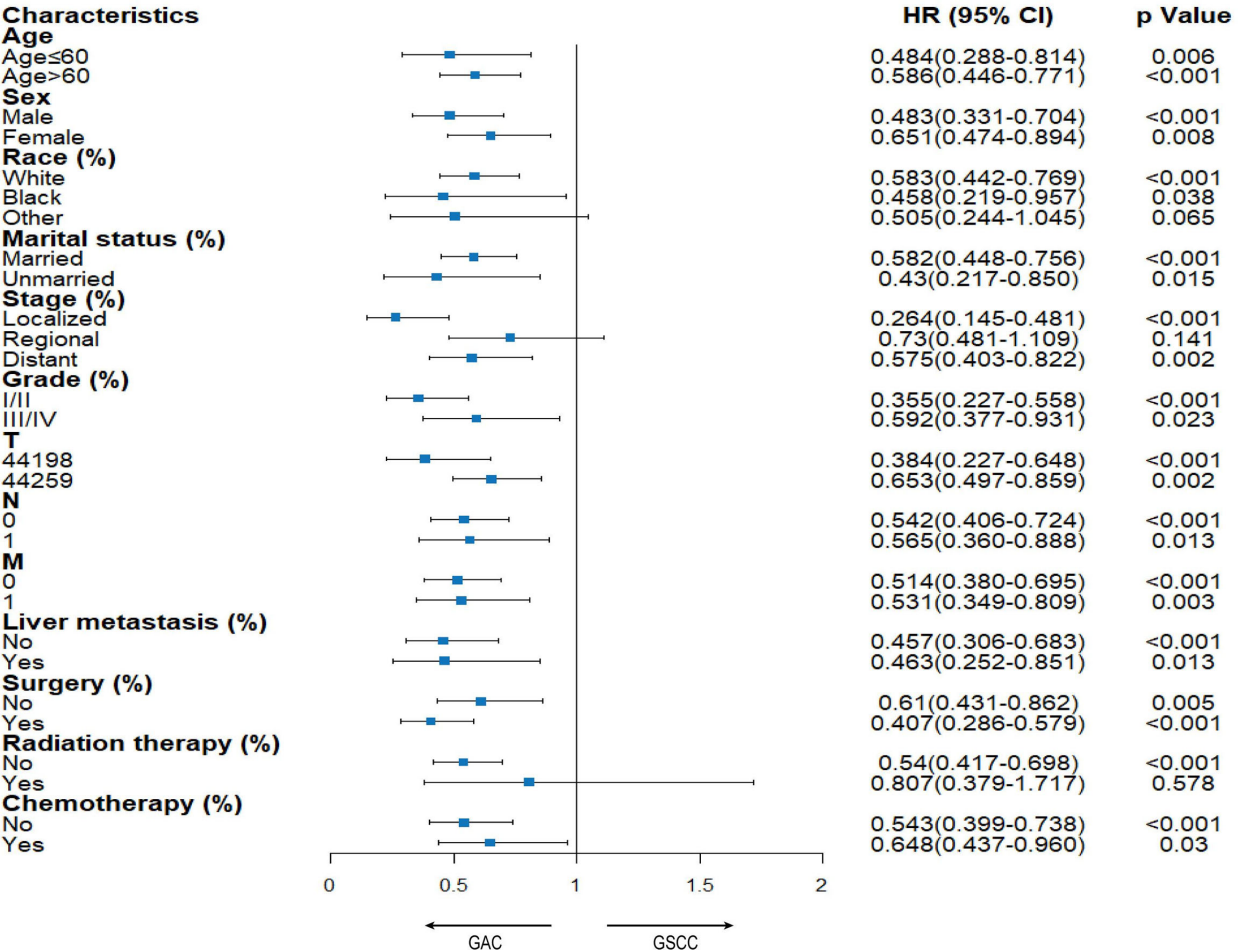
Stratified analysis between GAC and GSCC cohorts.

A subgroup analysis was conducted to compare OS between patients diagnosed with the above two tumors (**Figure 4**). There was a significant difference in almost all variables, indicating that the GSCC group was associated with a significantly poorer OS than the GAC group. Although in multivariate analysis (**Table 2**), there was no significant difference in radiation of the GSCC cohort, patients receiving radiation exhibited comparable outcomes among GSCC and GAC cohorts in the stratified analysis.

## Discussion

GSCC was a rare malignancy, accounting for less than 3% of all GBC (4).There was no accurate information on GSCC due to the rarity of this disease. In our study, the majority of GSCC patients were females, Caucasian, and age more than 60 years old worldwide. Furthermore, the GSCC cohort had a more distant-stage disease and a lower proportion of well-differentiated histology compared to the GAC cohort. Regarding treatment, GSCC patients could significantly benefit from surgery, chemotherapy, and radiation, which was similar to GAC cohorts.

Furthermore, we discovered that GSCC was correlated with poor prognosis. Compared to GAC, GSCC showed a higher SEER stage, higher grade, and a more advanced T-stage. Additionally, there were a lower proportion of patients who conducted adjuvant therapy like chemotherapy and radiation. It seems that these aggressive clinicopathological features and incomplete treatment may be the cause of the poor outcome of GSCC. We also used PSM to counterbalance the uneven clinicopathological features, and a significant difference in the prognosis between two subtypes was still observed. To our knowledge, no studies on GSCC have taken advantage of PSM. Additionally, a similar result can also be observed in stratified analysis for GSCC and GAC. Altogether, these results mean that GSCC had a worse outcome than GAC. However, a few studies have concentrated on molecular mechanisms of GSCC. To improve the prognosis of GSCC patients, there is an urgent need to conduct further fundamental and clinical studies to explore the potential molecular mechanism of GSCC.

A previous study conducted by Ayabe et al. (3) analyzed clinicopathological, and treatment characteristics between GSCC and GAC using another database, the National Cancer Database. In line with our study, they also concluded that patients with GSCC had a poorer outcome than patients with GAC (median OS, 9.0 months vs. 17.0 months; p < 0.001). However, they did not perform PSM to adjust the potential impact of different clinicopathologic values. In another study, Samuel et al. (13) also used the SEER database from 1988–2009 to compare clinicopathological characteristics and prognosis between different GBC histologic subtypes. They also demonstrated that SCC was associated with worse outcomes than other histological types covering papillary carcinoma and adenocarcinoma. But different from our study, they brought adenosquamous carcinoma (ASC) into SCC. This introduces the question of whether or not we should also consider ASC and SCC together. However, according to the World Health Organization’s Classification of Tumors and previous reports, ASC and SCC were different histological subtypes in the gallbladder (1, 7, 14). Therefore, based on the above reasons, we hold that ASC should not be considered together with SCC.

Besides gallbladder, adenocarcinoma (AC) and SCC was also compared in other locations like lung, esophagus, pancreas, cervix, rectum, and anus. A better OS of SCC was detected in the cervix (6, 15), rectum (16), and anus (17), while a worse OS of SCC was obtained in the esophagus (18) and pancreas (19). Current studies show different conclusions in survival advantages between AC and SCC of the lung, and it is difficult to differentiate whether prognosis is associated with pathologic patterns (20). Based on the above, we hold that SCC is not always related to worse clinical outcomes, which means histology is not the dangerous factor of prognosis, and location should also be considered.

There was also strength in this study. One is using data from the SEER database, which covers a wide geographic range of the United States, to provide a data set representative of the country’s diverse population. Another is using PSM and stratified analysis to counterbalance the potential influence of different baseline features. However, there are also some limitations in this study. First, vital information in the SEER database was not recorded. For example, patients treated with radiation or chemotherapy was associated with a better prognosis. However, records for some patients were unclear, which may underestimate the actual effect of treatment. Second, due to the rarity of GSCC, there were only small samples in the SEER database, which may result in selective bias. Consequently, prospective research with larger samples was needed to verify this conclusion.

## Conclusion

Majority of patients with GSCC were Caucasian, elderly females, which were similar to patients with GAC. Additionally, more than 50% of patients with GSCC and GAC have undergone surgery. However, patients with GSCC present worse outcomes compared to patients with GAC.

## Data Availability Statement

The data that support the findings of this study are available in Surveillance, Epidemiology, and End Results (SEER) Database 18 Regs (with additional treatment fields, 1975–2016 varying), using the SEER*Stat software (version 8.3.6; Surveillance Research Program, NCI, Bethesda, MD).

## Author Contributions

XC collected data, reviewed the literature and wrote the manuscript. YZ collected data, wrote and revised the manuscript. DP wrote and revised the manuscript. XS collected data and rechecked the manuscript. GW assisted in drawing. MQ designed and revised the manuscript. QX revised the manuscript. All authors contributed to the article and approved the submitted version.

## Conflict of Interest

The authors declare that the research was conducted in the absence of any commercial or financial relationships that could be construed as a potential conflict of interest.

## Publisher’s Note

All claims expressed in this article are solely those of the authors and do not necessarily represent those of their affiliated organizations, or those of the publisher, the editors and the reviewers. Any product that may be evaluated in this article, or claim that may be made by its manufacturer, is not guaranteed or endorsed by the publisher.
